# Dr. Thomas A. Dundas

**Published:** 1917-07

**Authors:** 


**Dr. Thomas A. Dundas,** N. Y. University 1878, died at his home in Elmira, May 27, aged 61.

